# Effect of thermocycling and surface treatment on repair bond strength of composite 

**DOI:** 10.4317/jced.53721

**Published:** 2017-08-01

**Authors:** Nazanin Kiomarsi, Pardis Saburian, Nasim Chiniforush, Mohammad-Javd Karazifard, Sedighe-Sadat Hashemikamangar

**Affiliations:** 1 Assistant professor, Department of operative dentistry, Dental school, Tehran University of Medical Sciences, International campus, Tehran, Iran; 2 Tehran University of Medical Science, International campus, Tehran, Iran; 3 Laser Research Center of Dentistry (LRCD), Tehran University of Medical Science, Tehran, Iran; 4 Department of epidemiology and biostatistics, Faculty of public health, Dental school, Tehran University of Medical Sciences, Tehran, Iran; 5 Associate professor, Department of operative dentistry, Dental school, Tehran University of Medical Sciences, International campus, Tehran, Iran

## Abstract

**Background:**

Repair of composite restorations is a conservative method that can increase the longevity and durability of restorations while preserving the tooth structure. Achieving a suitable bond between the old and new composite is difficult. To overcome this problem, some methods have been recommended to increase the repair bond strength of composite.This study aimed to assess the effect of aging by thermocycling (5,000 and 10,000 cycles) and mechanical surface treatments (Er,Cr:YSGG laser and bur) on repair shear bond strength of composite resin.

**Material and Methods:**

Totally, 120 composite blocks measuring 6x4x4 mm were fabricated of Filtek Z250 composite and were randomly divided into three groups (n=40) based on initial aging protocol: (a) no aging: storage in distilled water at 37°C for 24 hours, (b) 5,000 thermal cycles, (c) 10,000 thermal cycles. Each group was then randomly divided into two subgroups (n=20) based on mechanical surface treatment (laser and bur). The laser and bur-prepared surfaces were silanized and Adper Single Bond 2 was then applied. The repair composite was bonded to surfaces. Half of the samples in each subgroup (n=10) were subjected to 5,000 thermal cycles to assess durability of bond. The remaining half were stored in distilled water at 37°C for 24 hours and all samples were then subjected to shear bond strength testing in a universal testing machine with a crosshead speed of 1mm/min. Data (in megapascals) were subjected to one-way ANOVA and Tukey’s test (P=0.05). Mode of failure was determined under a stereomicroscope.

**Results:**

Bur preparation significantly improved the bond strength compared to laser (*P*<0.001). Aging by 10,000 thermal cycles significantly decreased the repair bond strength of composite (*P*<0.001). No significant difference was noted in this regard between distilled water and 5,000 thermal cycles groups (*P*=0.699). Primary bond strength and bond strength after 5,000 thermal cycles in the same subgroups were not significantly different either (*P*=0.342).

**Conclusions:**

Aging by 10,000 thermal cycles significantly decreases the repair bond strength of composite and surface preparation by bur provides a higher bond strength compared to laser.

** Key words:**Thermocycling, Composite, Repair, Laser.

## Introduction

Advances have been made in formulations of composite resins in the recent years. However, defects, chipping and fracture of composite restorations still occur. In the past, a defective composite restoration had to be replaced (1,2); however, this replacement was often associated with further loss of tooth structure and extension of the prepared cavity (3). Repair of composite restorations is a conservative method that can increase the longevity and durability of restorations while preserving the tooth structure (4). Advances in adhesives have enabled repair of old restorations instead of their replacement. This approach is conservative and cost effective because the intact part of the restoration remains untouched and thus, continuous irritation of dental pulp by removal of restoration can be avoided (5). However, in repair of composite restorations, it should be noted that changes that occur in composite resins over time due to aging including water sorption, chemical degradation and leaching of some compounds may decrease the reactivity of the remaining composite (old composite) and complicate the process of repair. This can even compromise the success of repaired restoration in some cases (6,7). In the process of repair, new composite is added to old composite. Since the old composite no longer has its unpolymerized superficial layer (oxygen inhibited layer), achieving a suitable bond between the old and new composite is difficult. To overcome this problem, some methods have been recommended to increase the repair bond strength of composite. The most commonly used method for this purpose is to increase the surface roughness via mechanical preparation followed by the application of silane and low-viscosity adhesives to help achieve a chemical bond (8). In fact, mechanical surface preparation aims to eliminate the outermost layer, provide a clean surface with high surface energy and create irregularities and porosities on the surface to increase the bonding surface area.

A recently suggested technique for this purpose is to use erbium lasers (9). Success of composite repair depends on duration and conditions of aging of old composite (duration of clinical service). These conditions should be taken into account by in vitro studies to simulate the clinical setting by artificial aging. Thermocycling is the most effective aging protocol. In this method, high heat in alternating cycles weakens the physicochemical properties of composite and decreases the number of unreacted double bonds on the surface and within the composite structure (10). Negative effects of thermocycling are influenced by the frequency of cycles, and the effect of 5000 thermal cycles on reduction of repair bond strength has been demonstrated in some previous studies (10,11). On the other hand, since 10,000 thermal cycles correspond to about one year of clinical service of composite (12), 10,000 thermal cycles are used to better simulate aging in the clinical setting.

This study aimed to assess the effect of surface treatment by laser and bur and frequency of thermal cycles on repair bond strength of composite. Also, in this study, the effect of thermocycling after bond was also evaluated to determine the durability of bond.

## Material and Methods

-Fabrication of composite blocks.

Totally, 120 composite blocks measuring 4x4x6 mm were fabricated of Filtek Z250 composite (A3 shade; 3M ESPE, MN, USA) in a plastic mold. Prior to application of composite, the mold was fixed on a glass slab. Composite was packed in the mold by a composite instrument. The composite block was light-cured from all directions for 40 seconds using VALO light curing unit (Ultradent Products, Inc., South Jordan, UT, USA) with a light intensity of 1000 mc/cm2 at 3mm distance. After removing the composite from mold, composite blocks were randomly divided into three groups (n=40).

1. Immersion in distilled water for 24 hours

2. 5,000 thermal cycles

3. 10,000 thermal cycles

All thermal cycles were performed between 5-55°C with 20 seconds of dwell time. Each group was then divided into two sub-groups (n=20) for mechanical preparations as follows:

1. Bur preparation: One surface of samples was roughened by diamond bur (835/008; Tees Kavan, Iran) using a high speed handpiece under water spray for three seconds. A new bur was used for preparation of every five blocks.

2. Preparation with Er,Cr:YSGG laser: One surface of each sample was irradiated by Er,Cr:YSGG laser (water lase, Biolase, Technology, san clement, CA, USA). Laser was irradiated in pulse mode with 2.78µm wavelength, 20Hz frequency and 3W po-wer for 10 seconds with back and forth motion perpendicular to the surface. The distance from the tip of laser handpiece (M28-6mm – Zip Tip- biolaser) to surface was 1mm. Laser was irradiated under water spray.

The surfaces of all samples prepared by laser and bur were etched with 37% phosphoric acid (Etch Royal, Pulpdent, Watertown, USA) for 15 seconds, rinsed for 15 seconds and completely dried with air spray. Silane (Angelus, Emergo Erupe, Metherland) was then applied by a microbrush on the surface of samples according to the manufacturer’s instructions for 10 seconds and after one minute, it was dried by air spray. Adper Single Bond 2 (3M ESPE, St. Paul, MN, USA) was then applied on the silane for 10 seconds and light-cured for 20 seconds. Repair composite (Filtek Z250; 3M ESPE, St. Paul, MN, USA) was then applied to a plastic mold measuring 3x3mm placed at the center of surface of each sample and light-cured for 40 seconds. After removing the mold, final curing of the samples was done for 20 seconds. All samples were stored in distilled water. Next, half of the samples in each subgroup (n=10) were subjected to 5000 thermal cycles between 5-55°C with 20 seconds of dwell time (for assessment of durability of bond).

-Shear bond strength testing.

Samples stored in distilled water at 37°C for 24 hours and those subjected to thermocycling underwent shear bond strength testing in a universal testing machine (Zwick Roell, Ulm, Germany) and load was applied to the interface of new and old composite resins by a blade at a crosshead speed of 1mm/minute until failure. Bond strength data were converted to megapascals and analyzed using one-way ANOVA and Tukey’s test.

-Assessment of mode of failure.

The fracture surfaces were evaluated under a stereomicroscope (Nikon, Tokyo, Japan) at x40 magnification to determine the mode of failure. Mode of failure was categorized as adhesive, cohesive or mixed (a combination of adhesive and cohesive). The study has been approved by the ethics committee of Tehran University of medical sciences.

## Results

**Figure 1:**
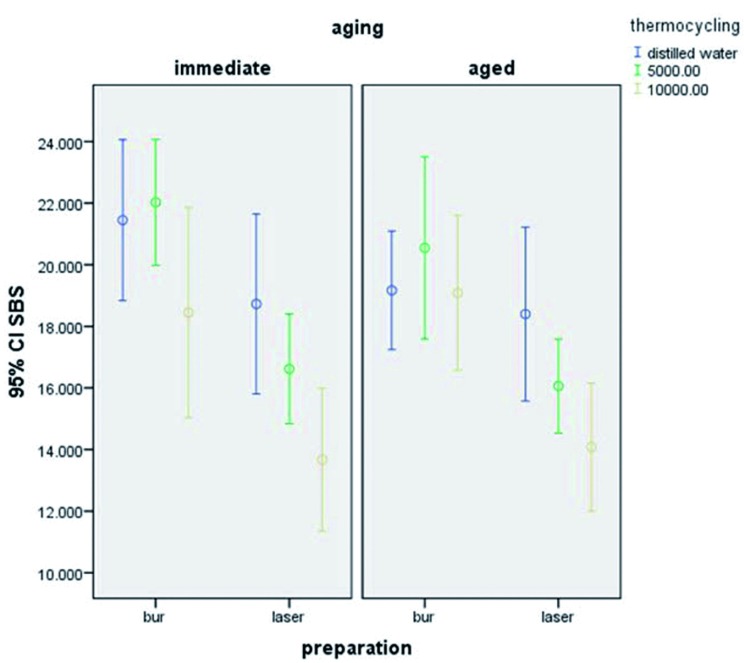
Shear bond strength in the study groups.

 Table 1 shows the mean and standard deviation of shear bond strength in the study groups. According to one-way ANOVA, sur-face preparation by bur and laser had a significant effect on bond strength (*P*<0.001). As shown in Figure 1, bur preparation had a greater effect on improving the bond strength compared to laser. Also, primary aging had a significant effect on bond strength (*P*<0.001). Tukey’s test was used for pairwise comparisons of the groups and showed no significant difference between distilled water and 5000 thermal cycles groups (*P*=0.699). However, significant differences were noted between distilled water and 10,000 thermal cycles (*P*=0.000) and 5000 and 10,000 thermal cycles groups (*P*=0.005) such that 10,000 thermal cycles significantly decreased the bond strength.

**Table 1:**
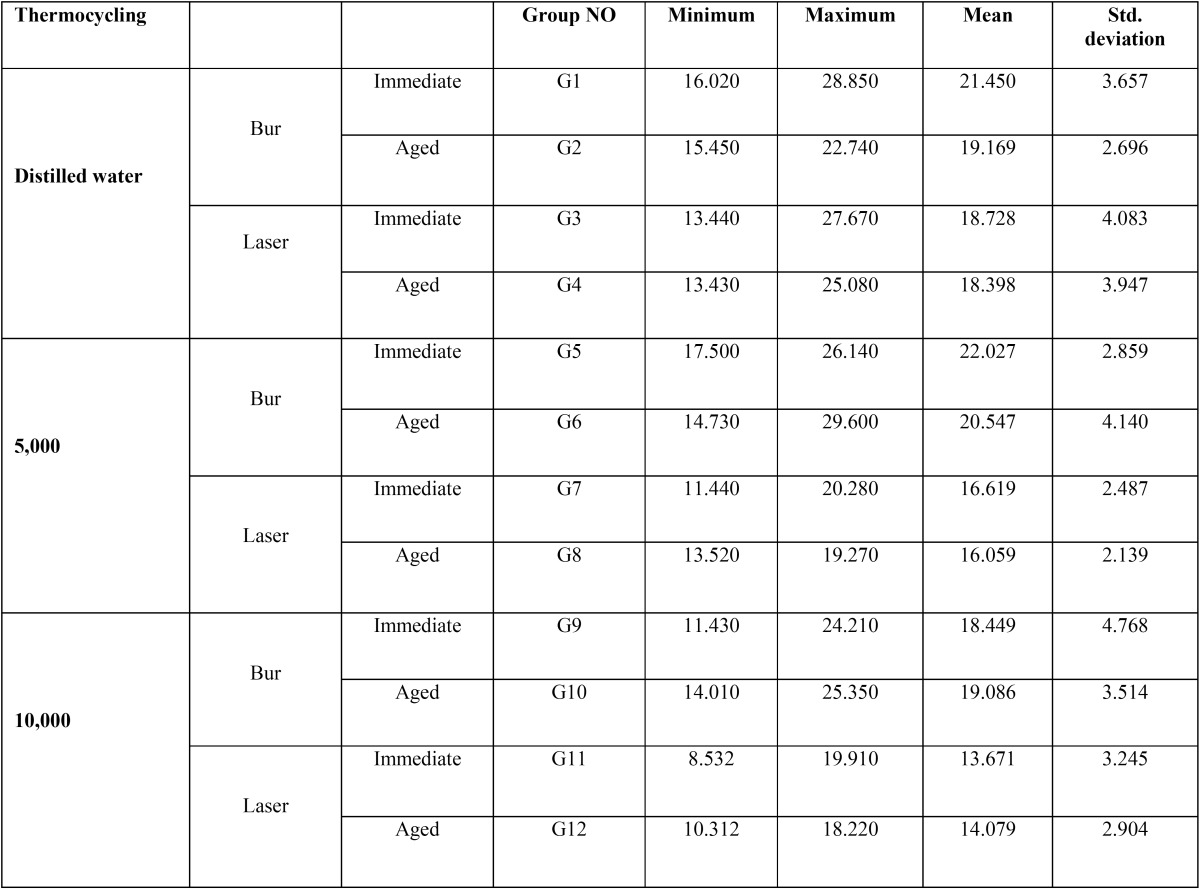
The mean and standard deviation of shear bond strength in the study groups.

As shown in Figure 1, significant differences were noted between bur and laser prepared groups with 95% confidence interval.

**Figure 2:**
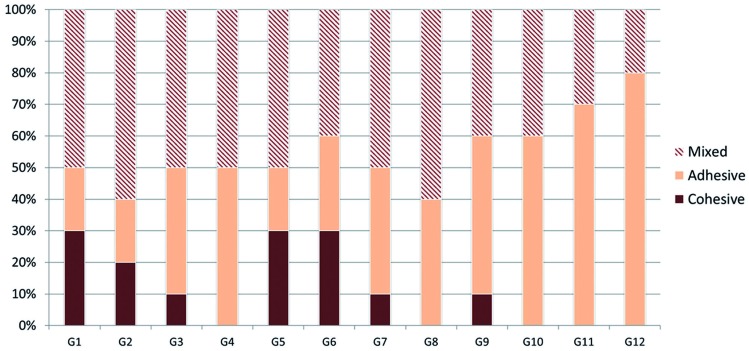
The frequency of modes of failure in the study groups (in percentage).

Figure 2 shows the frequency of modes of failure in the study groups (in percentage)

## Discussion

This study assessed the effect of aging by thermocycling and surface preparation on repair bond strength of composite resin.

Shear test was used to assess the bond strength, which is the most commonly used test for assessment of repair bond strength (13-17). This method has advantages such as easy preparation of samples and simple testing protocol (18). On the other hand, shear test simulates the oral clinical setting more efficiently than other tests in assessment of composite-composite bond (13,19). Repair of composite restorations is superior to complete replacement in terms of cost, accuracy and preserving the tooth structure (5).

Composite repair may be required a couple of months or years after the first restoration. Thus, some changes might have occurred in the primary composite restoration over time such as water sorption, chemical degradation and leaching of some constituents; these changes can negatively affect the repair process (20). On the other hand, age of restoration plays an important role in repair bond strength (21). There are still some discussions and questions regarding the efficacy and durability of repaired composites depending on duration of clinical service. Thus, the current study was performed to elucidate this topic.

Özcan *et al.* (10) stated that thermocycling is more effective than other methods for simulation of aging of composites and creates more challenging conditions for composite restorations. Thermocycling is performed aiming to create thermal strains at the bonding interface by thermal changes in water baths between 5-55°C. Repetition of thermal alterations in this process weakens the bond between resin matrix and filling material (22). Several factors in thermocycling can affect the bond strength test result such as temperature setting, dwell time and number of cycles. The latter is the most effective parameter in this respect (23). Thus, in this study, number of thermal cycles was considered as one of the variables. Özcan *et al.* (10) and Rinastiti *et al.* (11) showed that 5000 thermal cycles affect the bond strength of composites. On the other hand, Gale *et al.* (12) suggested that 10,000 thermal cycles (considering the fact that 20 to 25 thermal cycles averagely occur per day) correspond to one year of clinical service of composite restorations. Thus, number of cycles (5000 and 10,000) was considered as a variable in our study.

Free radicals in composite resins are responsible for adhesion between different composite layers. These free radicals decrease following aging. The greatest activity of residual free radicals of substrate occurs within 24 hours after composite polymerization (24). In this study, groups without aging which underwent repair process after 24 hours served as controls for the purpose of comparison and assessment of the effect of number of thermal cycles on adhesion in composites. Our results revealed that 5,000 thermal cycles did not have a significant effect on repair bond strength of composite, which was in line with the results of Magni et al. (25). However, Özcan *et al.* (10) and Rinastiti *et al.* (11) showed that this frequency of thermal cycles significantly decreased the repair bond strength of composite. Bektas *et al.* (26) demonstrated that 5000 thermal cycles decreased the bond strength only in samples prepared by bur and had no significant effect on bond strength of samples prepared by laser. The difference between our results and those of other studies may be due to several factors such as differences in the types of composite resins used, different surface treatment methods in repair process and different testing conditions.

Based on the current results, surface preparation by bur yielded higher bond strength compared to laser application. Both bur and laser increase surface roughness and free surface energy (27). However, differences in the results appear to be due to different patterns of surface roughness following the use of these methods (28). Tabatabaie *et al.* (29) mentioned that following the use of bur, macroretentive and microretentive areas are created, which enhance optimal bond while laser application mainly creates macroretentive areas, which was also observed in our study. Kimyai *et al.* (9) reported that repair bond strength of composite following surface preparation by laser was significantly higher compared to bur. One reason for the difference between our results and those of Kimyai *et al.* is the difference in laser parameters. We used 3W laser power while they used 2W laser. Evidence shows that laser parameters can significantly affect the bond strength (26,30), and the higher the energy and power of laser, the greater would be the destruction caused by laser irradiation. In other words, increasing the power of laser increases the diameter and depth of ablated area and micromechanical retention decreases as such (31). Laser irradiation can also cause separation of filler from matrix, which can negatively affect the bond strength (32). Lizarelli *et al.* (33) stated that difference in size of particles and composite resin composition can affect the volume and depth of ablation. On the other hand, Z250 hybrid composite was used in this study, which has lower cohesion level than other composites and is easily ablated following laser irradiation. Resultantly, destructive effects of laser would be greater on this composite compared to other composites (34).

On the other hand, our results revealed no significant difference in primary bond strength (after 24 hours) and bond strength after 5,000 thermal cycles (for assessment of durability of bond) in different groups. In fact, after repair, thermocycling negatively affects adhesion due to mechanical stresses applied to the bonding interface (due to difference in the coefficient of thermal expansion of materials) as well as hydrolytic degradation of hydrophilic elements in adhesives (35). In our study, 5,000 thermal cycles had apparently no significant effect on bond strength. Higher number of cycles or other aging methods such as water storage for a couple of months may negatively affect the bond strength. Another issue with regard to aging by thermocycling is that following thermal changes (to simulate aging), post-curing occurs at the bonding interface, which is actually beneficial and reverses the negative effect of thermocycling, i.e. hydrolytic degradation. In fact, post-curing and hydrolytic degradation are the positive and negative effects of thermocycling, respectively. Thus, absence of a significant difference between immediate bond strength and bond strength after thermocycling may be attributed to the positive effect of post-curing compensating for the negative effect of hydrolytic degradation (8). Teixeria *et al.* (36) stated that repair bond strength must be in the range of 15 to 25 MPa. In this study, most groups (except for groups 11 and 12) yielded optimal repair bond strength. The low mean repair bond strength in groups 11 and 12 was due to the negative interaction effect of high number of thermal cycles and laser application on bond strength. In the above-mentioned two groups, failure mode was mainly adhesive. Pairwise comparison of bond strength in the same subgroups (similar aging) revealed that laser treated groups had higher frequency of adhesive failure and lower frequency of cohesive failure compared to bur-prepared groups, which further highlights the superior efficacy of bur compared to laser for this purpose.

## References

[1] Mjör IA, Moorhead JE, Dahl JE (2000). Reasons for replacement of restorations in permanent teeth in general dental practice. Int Dent J.

[2] Papacchini F, Toledano M, Monticelli F, Osorio R, Radovic I, Polimeni A (2007). hydrolytic stability of composite repair bond. Eur J Oral Sci.

[3] Gordan VV, Shen C, Riley J, Mjör IA (2006). Two-year clinical evaluation of repair versus replacement of composite restorations. J Esthet Restor Dent.

[4] Bacchi A, Consani RL, Sinhoreti MA, Feitosa VP, Cavalcante LM, Pfeifer CS (2013). Repair bond strength in aged Methacrylate- and Silorane-based composites. J Adhes Dent.

[5] Baur V, llie N (2013). Repair of dental resin-based composites. Clin Oral Invest.

[6] Padipatvuthikul P, Mair LH (2007). Bonding of composite to water aged composite with surface treatments. Dent Mater.

[7] vanckerckoven H, Lambrechts P, Van Beylen M, Davidson CL, Vanherle G (1982). Unreacted methacrylate groups on the surfaces of composite resins. J Dent Res.

[8] Hamano N, Chiang YC, Nyamaa I, Yamaguchi H, Ino S, Hickel R (2012). Repair of silorane-based dental compositer: Influence of surface treatments. Dent Master.

[9] Kimyai S, Mohammadi N, Navimipour EJ, Rikhtegaran S (2010). Comparison of the effect of three mechanical surface treatments on the repair bond strength of a laboratory composite. Photomed Laser Surg.

[10] Ozcan M, Barbosa SH, Melo RM, Galhano GA, Bottino MA (2007). Effect of surface conditioning methods on the microtensile bond strength of resin composite to composite after aging conditions. Dent Mater.

[11] Rinastiti M, Ozcan M, Siswomihardjo W, Busscher HJ (2011). Effects of surface conditioning on repair bond strengths of non-aged and aged microhybrid, nanohybrid, and nanofilled composite resins. Clin Oral Investig.

[12] Gale MS, Darvell BW (1999). Thermal cycling procedures for laboratory testing of dental restorations. J Dent.

[13] Cho SD, Rajitrangson P, Matis BA, Platt JA (2013). Effect of Er,Cr:YSGG laser, air abrasion, and silane application on repaired shear bond strength of composites. Oper Dent.

[14] Brosh T, Pilo R, Bichacho N, Blutstein R (1997). Effect of combinations of surface treatments and bonding agents on the bond strength of repaired composites. J Prosthet Dent.

[15] Bonstein T, Garlapo D, Donarummo J Jr, Bush PJ (2005). Evaluation of varied repair protocols applied to aged composite resin. J Adhes Dent.

[16] Yesilyurt C, Kusgoz A, Bayram M, Ulker M (2009). Initial repair bond strength of a nano filled hybrid resin: effect of surface treatments and bonding agents. J Esthet Restor Dent.

[17] Tezvergil A, Lassila LVJ, Vallittu PK (2003). Composite–composite repair bond strength: effect of different adhesion primers. J Dent.

[18] Versluis A, Tantbirojn D, Douglas WH (1997). Why do shear bond tests pull out dentin?. J Dent Res.

[19] Ozcan M, Cura C, Brendeke J (2010). Effect of aging conditions on the repair bond strength of a microhybrid and a nanohybrid resin composite. J Adhes Dent.

[20] Ferracane JL (2006). Hygroscopic and hydrolytic effects in dental polymer networks. Dent Mater.

[21] Söderholm KJ, Roberts MJ (1991). Variables influencing the repair strength of dental composites. Scand J Dent Res.

[22] Rinastiti M, Zcan M, Siswomihardjo W, Busscher HJ (2011). Effects of surface conditioning on repair bond strength of nonaged and aged microhybrid, nanohybrid, and nanofilled composite resins. Clin Oral Investig.

[23] Amaral FL, Colucci V, Palma-Dibb RG, Corona SA (2007). Assessment of in vitro methods used to promote adhesive interface degradation: a critical review. J Esthet Restor Dent.

[24] Rathke A, Tymina Y, Haller B (2009). Effect of different surface treatments on the composite-composite repair bond strength. Clin Oral Investig.

[25] Magni E, Ferrari M, Papacchini F, Hickel R, Ilie N (2011). Influence of ozone on the composite-to-composite bond. Clin Oral Investig.

[26] Bektas O, Eren D, Herguner Siso S, Akin GE (2012). Effect of thermocycling on the bond strength of composite resin to bur and laser treated composite resin. Laser Med Sci.

[27] Kusgoz A, Ülker M, Yesilyurt C, Yoldas OH, Ozil M, Tanriver M (2011). Silorane-based composite: depth of cure, surface hardness, degree of conversion, and cervical microleakage in class II cavities. J Esthet Restor Dent.

[28] Batista RG, Kamozaki BBM, Gutierrez CN, Caneppele FMT, Torres GRC (2015). Effect of different surface treatment on composite repairs. J Adhes Dent.

[29] Hasani Tabatabaei M, Alizade Y, Taalim S (2004). Effect of various surface treatment on repair strength of composite resin. J Dent TUMS.

[30] Hatipoğlu M, Barutcigil Ç (2015). Effects of erbium and chromium doped yttrium scandium gallium garnet and diode lasers on the surfaces of restorative dental materials: A scanning electron microscope study. Nigerian Journal of Clinical.

[31] Duran İ, Ural Ç, Yilmaz B, Tatar N (2015). Effects of Er:YAG Laser Pretreatment with Different Energy Levels on Bond Strength of Repairing Composite Materials. Photomed Laser Surg.

[32] Moezizadeh M, Ansari ZJ, Fard FM (2012). Effect of surface treatment on micro shear bond strength of two indirect composites. J Conserv Dent.

[33] Lizarelli RF, Moriyama LT, Bagnato VS (2003). Ablation of composite resins using Er:YAG laser–comparison with enamel and dentin. Lasers Surg Med.

[34] Lizarelli RFZ, Moriyama LT, Pelino JEP, Bagnato VS (2005). Ablation rate and morphological aspects of composite resins exposed to Er:YAG laser. J Oral Laser App.

[35] Xie C, Han Y, Zhao XY, Wang ZY, He HM (2010). Microtensile Bond Strength of One- and Two-step Self-etching Adhesives on Sclerotic Dentin: The Effects of Thermocycling. Oper Dent.

[36] Teixeira EC, Bayne SC, Thompson JY, Ritter AV, Swift EJ (2005). Shear bond strength of self etching bonding systems in combination with various composites used for repairing aged composites. J Adhes Dent.

